# Near-Field Passive Wireless Sensor for High-Temperature Metal Corrosion Monitoring

**DOI:** 10.3390/s24237806

**Published:** 2024-12-06

**Authors:** Noah Strader, Brian R. Jordan, Oguzhan Bilac, Kevin M. Tennant, Daryl S. Reynolds, Edward M. Sabolsky, Ashley C. Daniszewski

**Affiliations:** 1 Lane Department of Computer Science and Electrical Engineering, West Virginia University, Morgantown, WV 26506, USA; nls0015@mix.wvu.edu (N.S.); daryl.reynolds@mail.wvu.edu (D.S.R.); 2 Department of Mechanical, Materials and Aerospace Engineering, West Virginia University, Morgantown, WV 26506, USA; brj00003@mix.wvu.edu (B.R.J.); oguzhan.bilac@mail.wvu.edu (O.B.); kmt0007@mix.wvu.edu (K.M.T.); 3 Oak Ridge Institute of Science and Education (ORISE), Oak Ridge, TN 37831, USA; ashley.daniszewski@netl.doe.gov; 4 National Energy Technology Laboratory, Morgantown, WV 26505, USA

**Keywords:** corrosion sensor, high-temperature sensor, metal oxidation, passive wireless, LC resonator

## Abstract

This work focuses on the fabrication and evaluation of a passive wireless sensor for the monitoring of the temperature and corrosion of a metal material at high temperatures. An inductor–capacitor (LC) resonator sensor was fabricated through the screen printing of Ag-based inks on dense polycrystalline Al_2_O_3_ substrates. The LC design was modeled using the ANSYS HFSS modeling package, with the LC passive wireless sensors operating at frequencies from 70 to 100 MHz. The wireless response of the LC was interrogated and received by a radio frequency signal generator and spectrum analyzer at temperatures from 50 to 800 °C in real time. The corrosion kinetics of the Cu 110 was characterized through thermogravimetric (TGA) analysis and microscopy images, and the oxide thickness growth was then correlated to the wireless sensor signal under isothermal conditions at 800 °C. The results showed that the wireless signal was consistent with the corrosion kinetics and temperature, indicating that these two characteristics can be further deconvoluted in the future. In addition, the sensor also showed a magnitude- and frequency-dependent response to crack/spallation events in the oxide corrosion layer, permitting the in situ wireless identification of these catastrophic events on the metal surface at high temperatures.

## 1. Introduction

Metal corrosion continues to be the leading cause of performance degradation of most products in transportation, manufacturing, building structures, energy production and storage, and chemical synthesis sectors. From numerous studies over the past seven decades, it was estimated that the direct costs of metal corrosion can account for nearly 3–4% of a country’s gross domestic product [1]. As reported by various corrosion experts, and reported recently by the World Bank, the global direct monetary cost of corrosion was near USD 6 trillion in 2021 [1,2]. As in many of these economic estimations, the stated global economic cost does not include all the indirect costs within the estimation, which may include other financial effects due to corrosion failures such as human, environmental, and productivity losses [3]. Metal corrosion can generally be defined as a change of the initial metal chemistry (and phase) to an alternative compound, which typically includes the formation of an unwanted metal oxide phase (but may also include the formation of other metal compound phases). This phase change results in an expected alteration in the physical properties, usually resulting in the deterioration of the original desired metallic properties.

There are many types (or mechanisms) of metal corrosion, such as pitting, erosive, crevice, concentration, and galvanic, where all of these common types usually occur near room temperature and occur due to multiple electrochemical reactions. A less common and less complex form of metal corrosion is direct atmospheric oxidation which typically occurs at accelerated rates at high temperatures. With the further implementation of high-temperature piping and heat exchangers within many industries, such as in aviation, manufacturing, oil/gas production and transport, solar concentrators, and energy conversion boilers (coal combustion and nuclear technologies), high-temperature metal oxidation corrosion kinetics is gaining higher importance to monitor for both productivity considerations and for all the indirect costs discussed above [4,5].

Many techniques over the past decades have been used to monitor metal corrosion, especially for the more common corrosion mechanisms that occur at ambient pressures and temperatures. The most common method is through visual inspection and surface property measurements, but these methods require high labor and time investments. There are various in situ (real-time) sensor monitoring technologies, such as mass loss (of representative coupons), acoustic, thermography, optical fiber, pulsed eddy current (PEC), eddy current pulsed thermography (ECPT), and electrochemical probe sensors (such as open circuit potential, linear polarization resistance, electrochemical noise, impedance spectroscopy, frequency modulation, potentiostatic/galvanostatic step polarization). These methods are generally operable only at lower temperatures and pressures [6,7,8]. In the case of high-temperature oxidation corrosion, there are very few technologies available for in situ monitoring due to accessibility constraints (due to insulation and containers), temperature effects on electronics and power sources, and environmental effects on the sensing mechanism. A general comparison of the various corrosion technologies can be observed in Table S1 in the Supplementary Materials.

The corrosion process is a mixture of interdependent chemical, mechanical, and thermal processes that are difficult to monitor, especially when the sensing mechanisms attempt to relate the change in the physical properties of the metal (and sensor material) in real time within challenging operational environments. This is especially true for corrosion observations at elevated temperatures, such as for energy conversion/generation systems (like coal boilers, gasifiers, and turbine systems), where the temperatures exceed 500 °C which will limit the use of semiconductor-based electronics and battery systems. Over the past decade, there has been much work in deploying a wireless sensor design mimicking the radio frequency identification (RFID) technology demonstrated over the past decades for simple, low-cost, and low- to no-power sensor components for sensing and tracking [9]. These RFID-based sensors maintain the capability of potentially operating in these harsh environments since the sensing and communication mechanism of the sensor can potentially be free of electronic components that are susceptible to thermal/atmospheric degradation. It must be clarified that RFID sensor technology can be separated into two main categories, integrated circuit (IC)-chip-based and IC-chip-less sensors. Any RFID sensor technology that contains an IC chip would not be capable of operation and communication at temperatures >160 °C (due to the limitations of the IC components) [9]. Therefore, only IC-chip-less sensors can be operable at higher temperatures, where these sensors are typically termed “passive” wireless sensors. The passive sensing component is typically composed only of an LC resonator that is patterned on an inert refractory substrate. All the materials used to fabricate the LC resonator must show thermomechanical, chemical, and microstructural stability in the sensing environment. The LC resonator serves a dual purpose, where it acts as both the sensing device and the communication antennae. Researchers over the past decade have demonstrated the use of a passive RFID sensor for various structural health monitoring applications at room to low temperatures [9,10,11,12,13,14].

One design aspect that needs to be considered for a passive RFID sensor is the operational frequency range. The frequency will dictate the sensing penetration depth and the possible communication distance to the interrogation (reader) antennae. The sensor can be designated as low-frequency (LF, <300 kHz), high-frequency (HF, ~0.3–100 MHz), and ultra-high-frequency (UHF, ~300–1000 GHz), where the lower frequency sensors are limited by lower penetration depth and communication distance [14]. The interrogation distance of the RFID sensor is important since it will dictate the resolution and accuracy of the sensor reading but also define the location required for the electronics for signal reading/processing. The interrogation distance is usually defined as near field and far field. Near-field sensors operate at close proximity to the antenna (typically within one wavelength or more specifically, <2D^2^/λ, where D is the largest dimension of the sensor). Near fields are characterized by complex electromagnetic fields used for precise measurements. Far-field sensors work at much greater distances, where the electromagnetic waves are stable, predictable, and primarily radiative. The most interesting passive wireless RFID sensors to monitor metal corrosion over the past decade were based on a near-field integration to maximize spatial resolution and limit environmental interference [15,16,17,18,19]. The operational frequency of these designs varied from LF to UHF, but the majority of these works utilized commercially available RFID tags as sensors which were placed directly onto the corroding metal. The results were quite encouraging, where a noticeable frequency shift and amplitude change were identified for the target peak as a function of corrosion time (even up to 12 months of monitoring). A few of these works were able to identify distinct corrosion differences as a function of sensor position [6], and some were able to define the presence of cracks within the material [17,18,19]. Interestingly, all of the works did not directly relate the sensor signal to the actual measured corrosion rates of the metal; they just indicated that visible corrosion was occurring but did not align with the actual corrosion kinetics. In addition, since many of these works utilized a commercial RFID tag, all the measurements were completed at room temperature or <160 °C.

Generally, all wireless RFID sensor technologies used to identify and monitor the corrosion and/or crack propagation within metals are limited to operation <200 °C due to either the inclusion of IC electronics or due to the oxidation and decomposition of the sensor components (for chip-less architectures). It must be noted that there are passive RF wireless sensors that successfully operate above this temperature to monitor temperature and pressure. Various researchers over the past decade showed passive LC resonator sensors used in the near field that could effectively measure the temperature of the environment up to 1200 °C at distances typically <1 cm [20,21,22,23,24,25,26,27,28]. These sensor technologies were fabricated by surface printing the LC sensor design using metals (Ag or more refractory Pt or Ni) or conductive ceramic (electroceramic) inks onto a thin ceramic dielectric substrate. The performance of these sensors was shown to be very accurate and repeatable. A few researchers also successfully demonstrated far-field sensor designs (such as a patch antennae design) composed of similar metals or conductive ceramic compositions to produce an LC design printed onto a dielectric ceramic substrate. These demonstrations could effectively measure temperature over larger distances (>10 cm) up to 1000–1200 °C [28,29,30]. None of these passive RF sensor designs were effectively applied to monitor corrosion and crack formation in metals above a temperature of 200 °C.

In this work, a near-field, high-frequency wireless corrosion sensor was designed and fabricated to simultaneously measure the temperature and corrosion rate of metals operating at high temperature. The main focus of the developed sensor was to in situ measure the actual corrosion rate of metals that were thermally oxidizing purely due to a gas–solid reaction. The passive wireless sensor was based on a planar printed LC design, where the sensor design would be printed on a ceramic dielectric support to produce the monolithic sensor. This monolithic sensor was placed (without permanent bonding) onto the surface of the corroding metal. Computational electromagnetic modeling was used to design the high-frequency sensor where the signal intensity and sensitivity were evaluated to predict the optimal geometry and material properties required for corrosion sensing. In order to quickly evaluate the applicability of the passive corrosion sensor, a copper (Cu) 110 series metal was chosen as the surrogate metal for this study to provide a candidate that would rapidly oxidize at the high evaluation temperatures. Metals of more high-temperature applications theoretically would be of more interest in this study, such as boiler metals 304H- and 347H-type stainless steel, but to more efficiently investigate the technology, the Cu metal was chosen due to its rapid and well-characterized oxidation behavior. The passive sensors were tested for oxidation between 50 and 800 °C. In addition, the sensors were also tested on imperfect Cu substrates with defined surface defects. These defects would exemplify imperfections that would initiate cracking and spallation events during operation and cooling. The intent was to deconvolute the sensor’s ability to differentiate between corrosion and mechanical failure events during operation.

## 2. Materials and Methods

### 2.1. Sensor Operation Principles

**Figure 1:**
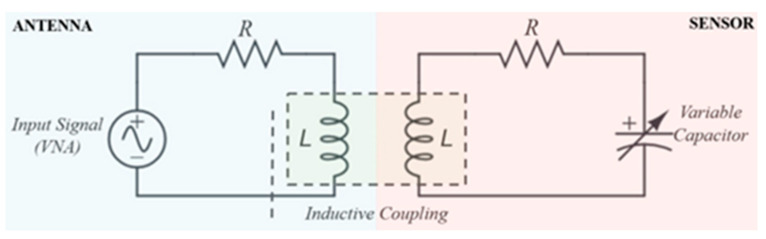
Schematic and interaction between the interrogator antenna (**left** half) and the sensor (**right** half). The resistance “R” is the resistance embedded in the combined materials.

The basis of this technology is a passive three-layer LC wireless sensor design. It has a top conductive inductor layer, a middle dielectric layer, and a bottom conductive ground plane layer. To communicate with the described sensor, an interrogator antenna that is connected to a source must wirelessly excite the sensor. The sensor then communicates through inductive coupling. The communication system can be described as an RLC circuit and is shown in Figure 1.

The top inductor layer of the sensor represents the inductor “L” on the sensor side of the schematic, while the loop in our loop antenna represents the inductor “L” on the antenna side of the schematic. Since the sensor has a dielectric layer between the inductor and the ground plane, this system also has an observed capacitance which is represented on the sensor side of the schematic as the variable capacitor. This sensor design is effective because it represents an RLC circuit, and every RLC circuit has a resonant frequency (f) which is given by the following [31]:

(1) $$f=\frac{1}{2\pi \sqrt{LC}}$$

In Equation (1), *L* and *C* are the inductance and capacitance of the resonator, respectively. Observing changes to this resonant frequency is how changes in temperature and corrosion are communicated. In addition to the resonant frequency, the quality factor *Q* of the resonator is also a consideration and is given by the following [31]:

(2) $$Q=\frac{1}{R}\sqrt{\frac{L}{C}}$$

In Equation (2), *R* is the embedded resistance in the resonator materials. A higher quality factor indicates a larger amplitude response when a resonator is coupled with a sinusoidally excited circuit. A higher quality factor also indicates a smaller signal bandwidth. This is ideal for this circuit because a large bandwidth is not necessary when information is communicated by tracking the resonant frequency. Therefore, this circuit is designed to have low resistance and capacitance and high inductance to maximize the quality factor.

The capacitance generated between the ground plane and the square planar inductor cannot be calculated by the general equation for capacitance between two plates because that assumes each capacitive plate is in a closed circuit. For this sensor, the loop antenna is connected to the vector network analyzer (VNA) through a closed circuit, but neither the inductor nor the capacitor is physically connected to the source or the ground. Therefore, they represent floating conductors, and the capacitance seen at the interrogator antenna can be expressed purely as the parasitic capacitance between them. This value is calculated primarily through simulation. Though the capacitance of the sensor cannot be calculated directly using an equation, it is still important to realize that the capacitance of any capacitor is still a function of the relative permittivity, which is the basis of the temperature sensing ability of the sensor. This equation is given by the following [32]:

(3) $$C=\frac{\varepsilon_{0}\varepsilon_{r}A}{d}$$

In Equation (3), $\varepsilon_{0}$ is the permittivity of free space (8.854 × 10^−12^ F/m), $\varepsilon_{r}$ is the relative permittivity of the dielectric material, *A* is the area of the capacitive plates, and *d* is the distance between the capacitive plates. The inductance is only a factor of the inductor shape. Therefore, it can be calculated directly. The inductance of the square planar inductor is given by the following [33]:

(4) $$L=\frac{1.27\mu_{0}n^{2}{\mathrm{d}}_{a\nu g}}{2}{\left[\ln\frac{2.07}{\rho }+0.18\rho +0.13\rho \right]}^{-1}$$

In Equation (4), $\mu_{0}$ is the permeability of free space (12.57 × 10^−7^ H/m), n is the number of turns of the inductor, ${\mathrm{d}}_{a\nu g}$ is the average diameter of the inductor coil, and ρ is the filling ratio which indicates what portion of space within the inductor corresponds to air or coil. ${\mathrm{d}}_{a\nu g}$ is given by the following [34]:

(5) $$d_{avg}=\frac{{\mathrm{d}}_{in}+d_{out}}{2}$$

In Equation (5), ${\mathrm{d}}_{in}$ is the inner diameter of the inductor, and $d_{out}$ is the outer diameter of the inductor. The filling ratio ρ is given by the following [34]:

(6) $$\rho =\frac{d_{out}-d_{in}}{{\mathrm{d}}_{in}+d_{out}}$$

The temperature and corrosion information are both communicated through different means. The sensor resonant frequency is a function of the relative permittivity of the dielectric layer, and this permittivity is a linear function of the temperature of its environment. Therefore, as the temperature increases, there is a direct effect on the resonant frequency of the sensor. The corrosion is communicated by the fact that the dielectric layer’s thickness increases as a corrosion layer is added between the dielectric and the ground plane at high temperatures, and the overall dielectric constant is changed by the mixture between dielectric materials in this layer. The addition of this layer changes the capacitance of the system, and the resonant peak of the sensor is directly affected by the system capacitance, as observed in the previous equations.

### 2.2. LC Resonator Modeling and Simulation

Models and simulations were created using the ANSYS HFSS 2023 R2 software, produced by ANSYS, Inc. (Canonsburg, PA, USA). The guiding principles for ANSYS modeling can be found in Table S2 in the Supplementary Materials [35]. This software uses electrical computer-aided design (ECAD) and mechanical computer-aided design (MCAD) modeling and translation to create 3D sensor models, and 3D low- and high-frequency static and transient solvers to characterize the sensor behavior. This behavior can be observed as 3D electromagnetic radiation (EM), surface currents, or the method used to communicate with the sensor in this study, “S parameters”, also referred to as “impedance parameters”. These parameters display reflected energy at various ports of the signal’s source and can be used to observe the resonant frequency of the sensor. For this study, the S11 parameter was observed. This parameter represents the energy reflected at the same port where the signal was propagated. This could also be referred to as S22 as it is a matter of port labeling. This parameter is used because if the source propagates a signal with the same resonant frequency as the sensor, a small amount of the propagated signal will be reflected to the port since they “resonate” with each other. This is the opposite if frequencies which are different from the sensor’s resonant frequency are propagated; a large amount of the propagated signal will be reflected to the port.

**Figure 2:**
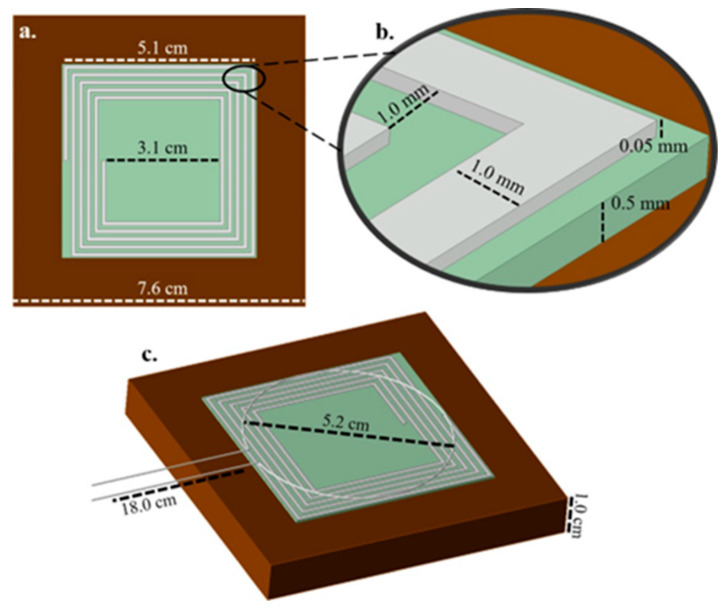
Three-dimensional geometry of sensor design produced in the ANSYS HFSS software. Shown in the figure is (**a**) the sensor top–down view, (**b**) the sensor close-up view of printed lines, and (**c**) and the sensor isometric view.

A schematic of the representative sensor and the interrogator antenna modeled to scale in ANSYS can be observed in Figure 2 in 3D dimetric and trimetric perspectives. All simulations were conducted using the Lumped Port method with a frequency sweep between 35 and 90 MHz to extract the S11 parameter of the sensor. A radiation boundary, designated with air as the material, was employed within the simulation environment, serving as a virtual enclosure to ensure the accurate computation of the EM radiation pattern. This boundary condition simulates wave propagation outward from the sensor, effectively capturing the interaction of the sensor with the surrounding space. The sensor is in the form of a planar inductor printed onto a dielectric substrate. The sensor is then set onto the corroding metal which acts as a ground plane. Therefore, the entire sensor system can be itemized and modeled using these three components: inductor, dielectric, and ground plane. The planar inductor was a square planar 5-turn coil. The material used for this inductor was silver (Ag) due to its high conductivity allowing for a larger received signal magnitude. The line width of the inductor was 1 mm, and the line spacing was 1 mm. The thickness of the inductor lines was 0.05 mm, and its inner diameter was 3.1 cm, while its outer diameter was 5.1 cm. This inductor was printed on top of a piece of square planar alumina (Al_2_O_3_) with a side length of 5.11 cm and a thickness of 0.5 mm. The ground plane used for the sensor was a square planar piece of copper with a side length of 7.6 cm and a thickness of 1 cm. This described sensor was interrogated by a platinum single-loop antenna fabricated entirely out of 0.2 mm thick platinum wire and was connected to a standard SMA connector. The loop diameter was 52 mm, and it had an 18 cm transmission line length from the base of the loop to the SMA connector. All of the material properties used in the ANSYS model can be observed in the Supplementary Materials (Table S3). A further description of the ANSYS building workflow can be found in the Supplementary Materials (Table S4).

Various sensor designs were simulated using this software with various levels of capacitance and inductance present in the sensor through different means such as line thickness, line spacing, additional capacitive plating of various shapes and sizes, and the number of turns on the sensor. Table 1 summarizes the different sensor components that were studied to determine the optimal design. The criteria considered for high performance were magnitude and frequency shifting in response to variation in the alumina dielectric, which corresponds to temperature change, and the addition of and variation in oxide layer thickness. Row (a) displays the horizontal print widths evaluated for the inductor lines. Row (b) shows the different thicknesses evaluated for the inductor lines. Row (c) shows the distance between each adjacent printed inductor line. Row (d) displays the size scale of the entire sensor including all components such as the inductor, dielectric, and ground plane. Row (e) displays the number of inductor sides (such as 3 being triangular-shaped and 4 being square, etc.). Row (f) shows the number of turns or loops for the inductor.

The baseline sensor design was a 1.0 mm line width, 50 µm electrode height, 1.0 mm line spacing, 100% sensor scale, 4-sided inductor, and 5 inductor turns. While one parameter was varied (of those shown in Table 1), the other parameters were held constant at these values. Therefore, every possible sensor combination of variables listed in this table was not simulated; each variable was isolated, and its effect on the sensor wireless response was observed individually.

Through repetitive simulations, an optimal design was chosen that provided the best sensitivity to both temperature changes and corrosion changes. In other words, the optimal model provided the largest overall received signal magnitude so that the sensor signal could be tracked clearly in the presence of noise and also the largest magnitude of the frequency shift that occurs through temperature variation and oxidation accumulation. The oxidation growth of the sensor was modeled by adding a layer of copper oxide (only CuO was considered) to the model that corresponds to the oxidation layer that would grow on the sensor when exposed to a temperature of 800 °C for 4 h. This exact layer thickness was determined through the corrosion kinetics of the copper ground plane. This copper oxide layer was then added to the model between the copper ground plane and the dielectric layer in equal increments up to the maximum thickness.

### 2.3. Sensor Fabrication

The sensors were fabricated using dense alumina (Al_2_O_3_) substrates (96% purity, MTI Corporation, Richmond, CA, USA) and Ag-based inks. The Ag ink was synthesized by using two Ag powders purchased from Beantown Chemical (Hudson, NH 03051, USA). The fine Ag particle size was 0.7–1.3 µm (99.9% purity, Lot#D06W05), and the coarse Ag particle size was 3–7 µm (99.9% purity, Lot#N01D020); both powders were used as received from the manufacturer. The Ag ink was formulated with fine and coarse Ag powders with a 1:4 ratio, respectively. The Ag powder mix was then dispersed into a Johnson Matthey ethyl cellulose and terpineol organic vehicle (Johnson Matthey, Smithfield, PA, USA), and two drops of fish oil (Tape Casting Warehouse, Morrisville, PA, USA) were added to aid in dispersion. The total mixture was then sonicated for 2 min. The LC sensors were then fabricated via a screen-printing process. A 230-mesh screen with our LC design was purchased from UTZ Technologies (Little Falls, NJ, USA). Three layers of Ag ink were printed in total with a 3 min time interval between layers to dry the previous layer. The fully printed sensor was then sintered in a box furnace (Sentry Xpress 4.0, Paragon Industries L.P., Mesquite, TX, USA) in ambient air. The sintering protocol was as follows: fired to 825 °C for 4 h with 2 °C/min for both the heating rate and the cooling rate. The final sintered sensor can be seen in the Supplementary Materials (Figure S1).

### 2.4. Copper 110 Preparation

A large Cu plate (6.35 mm in thickness (0.25 in)) of multi-purpose-grade 110 Cu (99.5% Cu, 0.5% O, McMaster Carr, Elmhurst, IL, USA) was used as the ground plane. The large plate was cut to the required dimensions (7.62 × 7.62 cm^2^ in area (3 × 3 in^2^)) followed by the polishing of the Cu surface to standardize the surface. The surface was abraded with multiple SiC papers of 400, 600, 800, 1200, and 1500 grit. The copper was then cleaned with DI water and dried before testing.

### 2.5. Passive Wireless Sensor Experimental Layout and Orientation

The silver 5-turn inductor sensor was printed on alumina, and the prepared Cu ground planes discussed in the previous sections were used for the experimental procedure introduced in this section. Nine identical experiments were conducted using three polished ground planes and three polished surfaces with 2 and 4 diamond saw cuts on the surface representing pre-existing surface defects. This will be discussed further in the following sections.

The experiment was broken into three thermal stages: the heating stage, isothermal hold, and cooling stage. For the heating stage, the furnace temperature was increased at a constant rate of 120 °C/h. The furnace was then held at a maximum temperature of 800 °C (isothermal hold) for 4 h. After this, the furnace temperature was decreased by −120 °C/h until it returned to room temperature.

**Figure 3:**
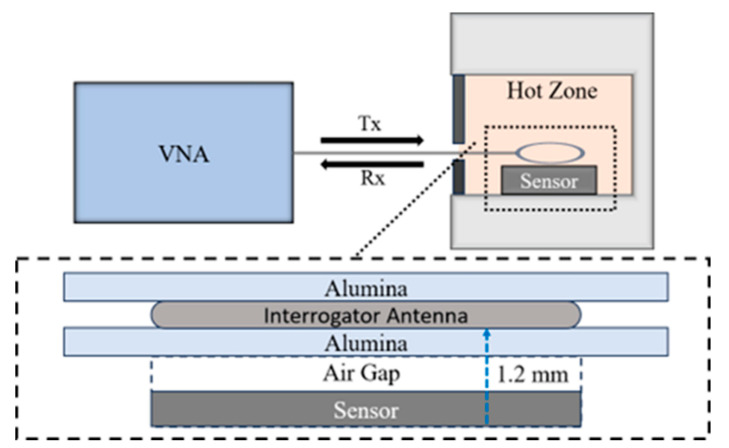
Antenna and sensor experimental setup displaying orientation of VNA, interrogator antenna, and sensor. It also displays the close-up view of the sensor and interrogator antenna orientation.

The sensors were placed directly in the middle of the furnace with the interrogator antenna placed directly above the center of the sensor at a distance of 1.2 mm. The interrogator antenna was a single-loop antenna constructed out of a single 0.2 mm platinum wire. In addition to the interrogator antenna, a square 0.5 mm thick plate of alumina with an area of 26 cm^2^ was placed above the sensor at a distance of 0.7 mm. The alumina plates were 96% dense with a purity of 99% (MTI Corp., Richmond, VA, USA). The interrogator antenna loop rested directly on this alumina plate to maintain a consistent distance from the sensor during the heating and cooling cycles of the experiment where sensor droop is prone to occur due to the malleability of the metallic interrogator antenna. In addition to this alumina plate, a second alumina plate with the same dimensions was placed on top of the interrogator antenna loop to ensure the interrogator antenna did not undergo any deformation during the heating and cooling cycles and remained flat. These alumina plates were added with no physical connectors such as hardware or adhesives to either the interrogator antenna or the sensor itself. All of the components were connected by resting them upon each other. Placing an alumina plate between the interrogator antenna and the sensor does not affect the signal received from the sensor. A schematic of the corrosion experiment setup used is shown in Figure 3.

The sensor S11 response was captured using a Keysight Technologies N9918A 26.5 GHz FieldFox Microwave Analyzer (Santa Rosa, CA, USA) controlled with MATLAB R2023b (MathWorks, Natick, MA, USA). This parameter was observed as the frequency (MHz) versus LOG-magnitude (dB) of the S11 parameter of the sensor over a frequency range of 50–125 MHz at the maximum output power of 1 dBm. dBm is a common unit of power used in radio technology and represents the power across the full frequency sweep for each measurement in this experimental procedure. The frequency response was captured every 30 min during the heating and cooling stages and every 20 min during the four-hour max temperature hold. Each capture comprised 100 captures separated by three seconds, and these 100 captures were averaged in MATLAB in post-processing to reduce the presence of outliers and reduce the power of noise in the received signal. Three sensors were tested at each temperature condition, but consistent measurements for only the 800 °C tests were obtained, and the average response for this measurement is shown in the following sections. The reason for the varied responses at temperatures <800 °C due to crack formation is discussed in the below sections.

## 3. Results and Discussion

### 3.1. Corrosion Kinetics of Cu 110 Grade

**Figure 4:**
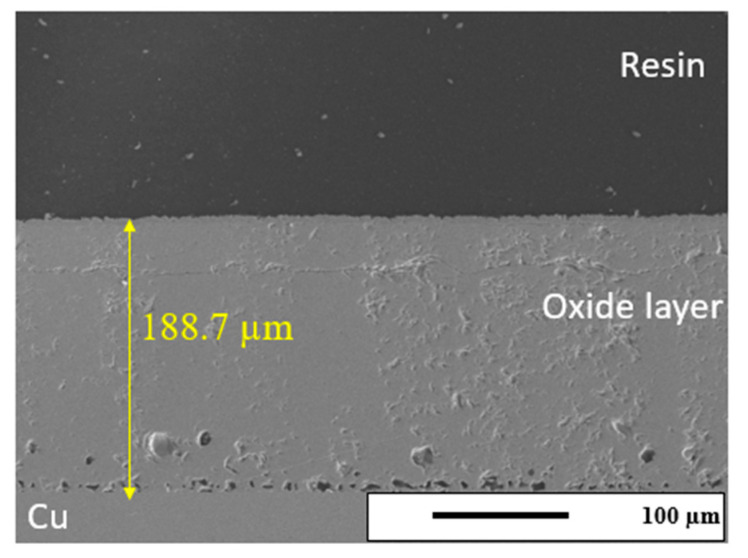
SEM cross-sectional image of oxide layers on copper oxidized under air atmosphere for 4 h at 800 °C.

Previous researchers studying the oxidation of copper showed that the oxidation rate follows a parabolic time law dependence, but slight variations in this fit were discovered in the range of 350–1050 °C [36,37,38,39,40]. Based on these discrepancies, three distinct regions were identified: high temperature (900–1050 °C), intermediate temperature (600–900 °C), and low temperature (350–600 °C). Knowing this, and the slight variations shown in each work over the past decades, before initiating the sensor tests on the Cu ground planes in the current work, the oxidation kinetics for the Cu 110 used in these experiments were characterized. One goal of this current work is to relate the oxidation kinetics directly to the sensor signal, which has not been accomplished in previously published works. Table S5 (in the Supplementary Materials) shows the oxidation kinetic constant, typically termed k” (in g^2^ cm^−4^ s^−1^), measured for the Cu 110 by isothermal TGA (Discovery TGA, TA Instruments, New Castle, DE, USA) measurements at 600, 700, and 800 °C. The k” values for 600, 700, and 800 °C were measured as 2 × 10^−8^, 5 × 10^−8^, and 1 × 10^−7^ g^2^ cm^−4^ s^−1^, respectively. The activation energy calculated from the slope of the graph (Figure S2) was 76.6 kJ/mol with an R^2^ fit parameter of 0.9947. In the literature, the activation energy of copper in an air atmosphere between 600 and 800 °C was found to be 27–123 kJ/mol [38,40,41,42,43,44]. This study’s activation energy value (76 kJ/mol) is within the previously found range. Figure S3 (in the Supplementary Materials) shows the estimated oxide layer thickness calculated from the TGA weight kinetics data, using the surface dimensions of the samples. These data will be later used to compare against the sensor signal during oxidation. Figure S3a displays the estimated oxide thickness growth on heating to 800 °C, and Figure S3b shows the growth during the isothermal hold at 800 °C. In order to collaborate the kinetic trends estimated from the TGA data, slices of Cu 110 were also oxidized in an air furnace using the same heating and isothermal hold conditions. Small cuts of the Cu 110 with dimensions of 1 × 25.4 × 6.35 mm were polished with the previously stated polishing protocol to prepare the SEM samples. The samples were then fired to 800 °C for 4 h with a heating and cooling rate of 2 °C/min. Figure 4 displays the SEM (JOEL JSM-7100F, Peabody, MA, USA) image of the cross-section of one of the samples held at 800 °C for 4 h. From the SEM micrograph, it can be seen that the oxide–copper interface was shown to be uncracked and conformed to the roughness of the copper surface. The SEM micrograph also showed a line of porosity following this interface. The oxide thickness was measured to be ~189 µm from this micrograph. From the previously shown estimated oxide thickness kinetics (Figure S3a,b), the overall thickness change on heating and through the isothermal hold (the summation of Figure S3a,b) was found to be relatively well correlated. Therefore, the oxidation kinetics measured for Cu 110 can be directly related to the frequency signal shift measured by the wireless sensor (which will be further discussed in a later section).

### 3.2. ANSYS HFSS Sensor Modeling

To simulate the effects of increasing the temperature of the sensor’s environment, the sensor geometry was constructed in ANSYS HFSS, and the material composition of its layers was assigned using the material properties listed in Table S3. The sensor was then placed in an ideal airbox. The only variation introduced was the relative permittivity of the dielectric material, Al_2_O_3_, which was varied from 9.8 to 11. These values are shown in Table S3 and represent the material’s relative permittivity at room temperature and 800 °C.

**Figure 5:**
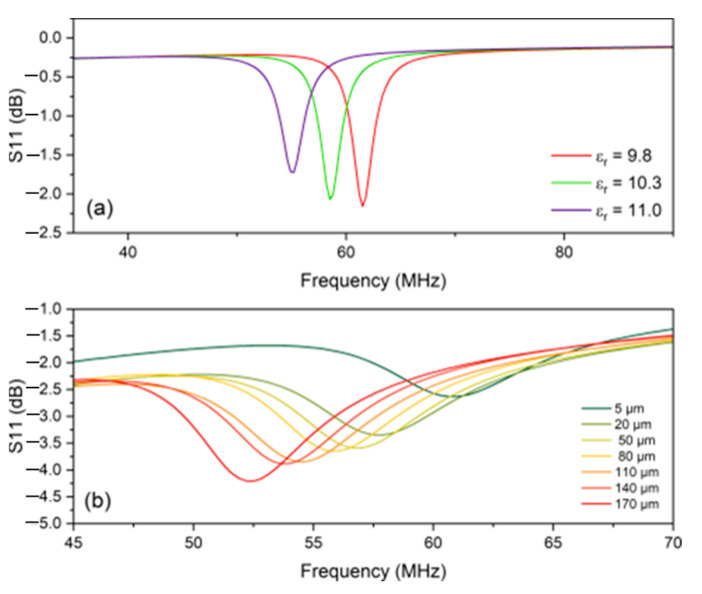
(**a**) ANSYS HFSS simulation results of LC sensor design by varying the relative permittivity of the Al_2_O_3_ dielectric layer from 9.8 to 11. (**b**) ANSYS HFSS simulation results of LC sensor with an increase in the copper oxide layer between the ground plane and the dielectric layer of the simulation from 5 to 170 µm.

Figure 5a displays the ANSYS HFSS simulation results of the sensor with the relative permittivity of alumina at 9.8 which represents the alumina at room temperature, 10.3 which represents the alumina at 400 °C, and 11 which represents the alumina at 800 °C. As the relative permittivity of the dielectric material increased from 9.8 to 11, the resonant frequency of the sensor decreased from 62 MHz to 55 MHz with a mostly linear relationship. This is the basis of the sensor’s temperature sensing ability and is related to Equation (3), which shows a proportional linear relationship between the capacitance of the sensor and the relative permittivity of the dielectric material. In addition to the frequency shift, the magnitude of the frequency response also attenuates (decreases in magnitude) slightly. This is due to the change in the capacitance of the system, as capacitance is directly related to the quality factor given in Equation (4), which is inversely proportional to capacitance. This result provided the foundation for the temperature sensing principle of the sensor and provided a method to validate the experimental performance of the sensor.

The effect of oxidation on the ground plane was observed through simulation by adding a uniform layer of copper oxide (CuO) between the ground plane and the alumina dielectric of the sensor. This layer had the same constant length and width as the ground plane (7.6 cm × 7.6 cm) but showed a variable thickness. The maximum copper oxide thickness estimated in these initial simulations at 800 °C for 4 h was 170 µm. This was determined through the corrosion kinetics borrowed from the literature initially [36]. This oxidation layer was added to the sensor at a minimum of 5 µm and was increased over seven increments to a maximum thickness of 170 µm. The results of this increasing thickness are observed in Figure 5b, which shows the ANSYS HFSS simulation results of the sensor with increasing copper oxide layer thickness.

As the thickness of the copper oxide layer increased, the magnitude of the S11 response increased from −2.5 dB to −4.25 dB, and the frequency shifted from 61 MHz to approximately 52 MHz. Both of these parameters have a near-linear relationship with the oxidation thickness. This simulation represents the physical characteristics of the sensor during the max temperature hold and gives evidence that the oxidation layer on the sensor’s ground plane has a trackable relationship with the frequency and magnitude of the S11 response. Since the sensor does not represent a closed-circuit model, the capacitance of the sensor seen at the interrogator antenna cannot be calculated nor validated by Equation (3), as discussed previously in this work. Therefore, this simulation result provides the best indicator of sensor performance during oxidation development and will be used to validate the sensor behavior during the experimental trials.

### 3.3. Passive Wireless Temperature and Corrosion Sensing at High Temperature

This section displays the results obtained from the wireless testing of the proposed wireless passive sensor design. The experiment consisted of a five-turn silver inductor printed on an alumina dielectric on three different variations of the copper ground plane. Each ground plane had the same dimensions but with different imperfections (cuts) added to the ground plane to demonstrate the sensor’s wireless response with different levels of structural health (SH). One ground plane had no surface defects, one ground plane had two perpendicular cuts dividing the copper ground plane into four equal sections (minimal surface defects), and one ground plane had four cuts dividing the copper ground plane into nine equal sections (maximum surface defects). The sensor’s wireless response was captured using the VNA during the heating and cooling stages to monitor the sensor’s temperature sensing ability and also during the max temperature 4 h hold to monitor the oxidation sensing ability.

**Figure 6:**
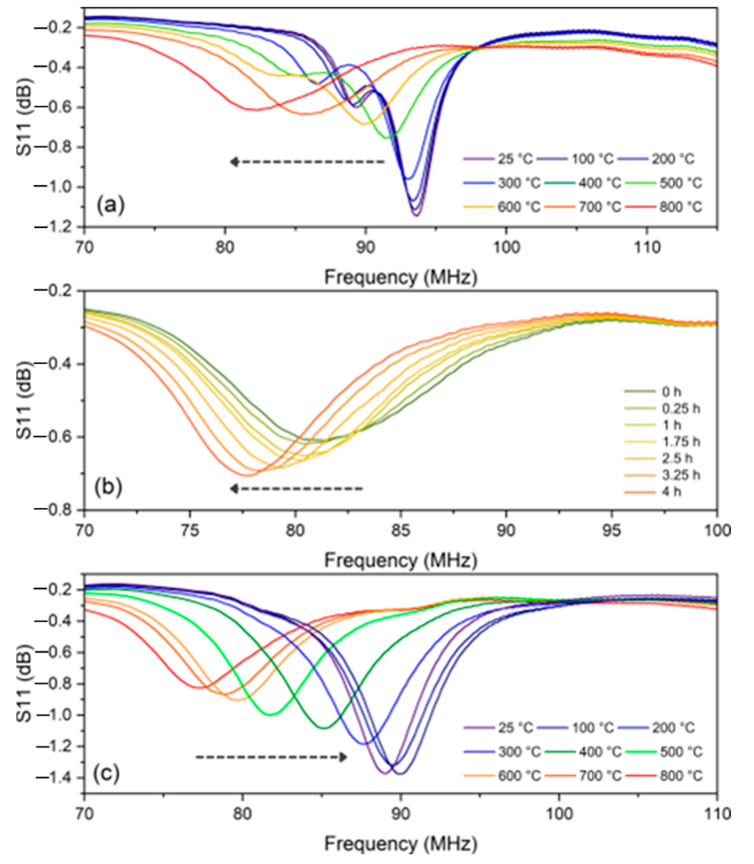
Passive wireless sensor heating and cooling results of the copper ground plane with no surface defects. (**a**) Sensor data for the heating stage from room temperature to 800 °C at a rate of 120 °C/h. (**b**) Sensor data at 800 °C isothermal hold for 4 h. (**c**) Sensor data for the cooling stage from 800 °C to room temperature at a rate of −120 °C/h.

Figure 6 displays the passive wireless sensor heating, cooling, and max temperature hold results for the ground plane with no added surface defects. During the heating stage in Figure 6a, the sensor’s peak decreased in magnitude by approximately 0.5 dB and shifted downward in frequency from 93 MHz to 82 MHz. This was the expected result because as the sensor was heated, the dielectric constant of the alumina increased. This caused a corresponding downward frequency shift and magnitude decrease. These results were in agreement with the simulation results observed in Figure 5a. Also, as the metallic material conductors increase in temperature, their conductivity decreases. This also causes an attenuation.

During the max temperature hold stage in Figure 6b, the sensor’s peak increased in magnitude by approximately 0.12 dB and shifted downward in frequency from 82 MHz to 76 MHz. These results were also in agreement with the trend in the simulation results observed in Figure 5b displaying that an increasing oxide thickness on the ground plane of the sensor causes the magnitude and frequency of the sensor response to increase and decrease, respectively, in a mostly linear fashion. The cooling stage of the ground plane with no defects is shown in Figure 6c which displays the inverse of the heating stage. It increased in magnitude by approximately 0.5 dB and had an upward frequency shift from 76 MHz to 89 MHz. This is also the expected result, as the dielectric constant of the alumina is increased during heating and is decreased during cooling.

**Figure 7:**
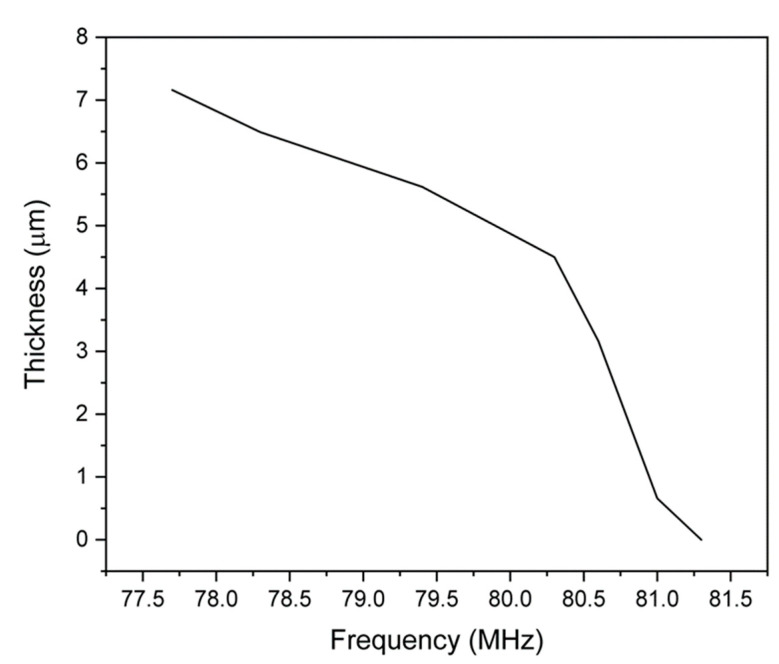
Copper oxidation growth thickness as a function of wireless corrosion sensor signal frequency at 800 °C for a 4 h isothermal hold (after heating to 800 °C at 2 °C/min in air).

As seen in Figure 6, the frequency and magnitude response during the heating and cooling stages showed a very predictable trend, and the magnitude and frequency variation are consistent during heating and cooling. The trend during the max temperature hold is also predictable with little to no variation or outliers. This oxidation growth provides an offset between the initial and final sensor frequency, making oxidation tracking trivial in this case. This frequency and magnitude offset can be related to the exact oxidation thickness accumulated on the ground plane by comparing the observed frequency shift at a certain time with the corrosion kinetics obtained previously. Figure S4 shows the oxide growth thickness (derived from the isothermal TGA data) and sensor frequency shift data extracted from the main peak in Figure 6 during the isothermal hold at 800 °C for 4 h. During the isothermal hold stage, the oxide layer thickness increased by ~7 microns, and the frequency shifted from 81.3 MHz to 77.7 MHz, which is derived from the oxidation thickness growth on the Cu ground plane. Therefore, a correlation of the oxidation kinetics and sensor data plot results in a calibration trend for the corrosion sensor. This calibrated relation for the corrosion sensor (at 800 °C) is shown in Figure 7. It must be noted that this relation only holds when the Cu 110 sample is prematurely heated to 800 °C at a rate of 2°/min in air since there was a premature oxidation that occurred in the sample during this heating. This amount (thickness) of premature oxidation is shown in the TGA kinetic data in Figure S3a. Future investigation will be required to relate the sensor frequency to the current and previous oxide thickness growth and the relative temperature (and atmosphere) of the metal environment.

### 3.4. Passive Wireless Sensing of Cracking and Spallation of Corrosion Layers

**Figure 8:**
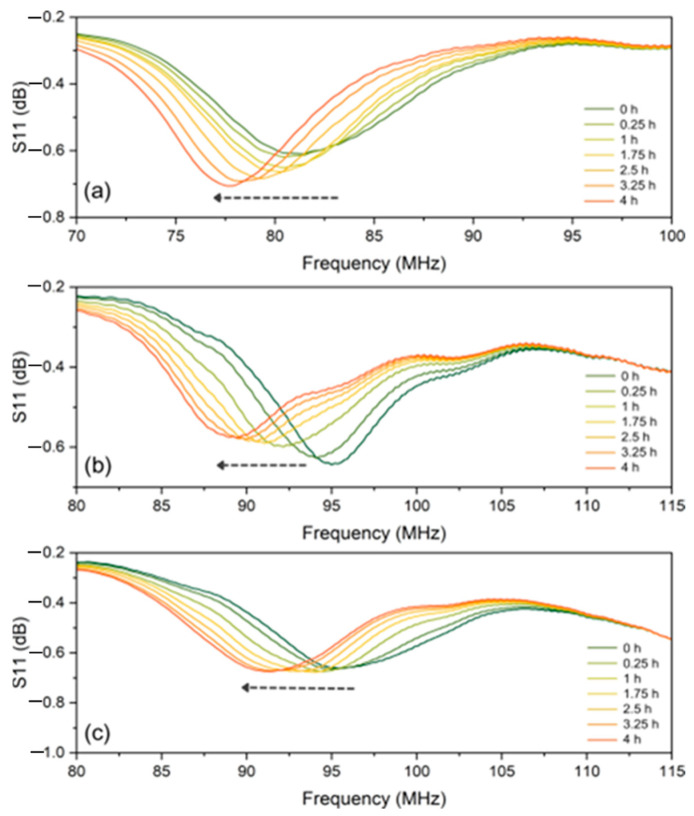
Passive wireless sensor response for the Cu oxidation during the isothermal hold at 800 °C for ground planes with different levels of pre-existing surface defects. (**a**) Defect-free ground plane, (**b**) minimal defects in the ground plane, and (**c**) maximum defects in the ground plane.

One important observation from completing the multiple trials at 800 °C was the appearance of large magnitude jumps in the signal amplitude during the cooling segment. These results did not conform to the previous trend lines. It was then observed when the ground plane was removed from the furnace that the oxide layer had experienced significant spallation and cracking. From these results, it was hypothesized that the formation of cracks or delamination in the oxide layer would cause a shift in the signal frequency and decrease in the amplitude. To verify this, additional ground planes with added surface defects were tested to increase the probability of spallation and cracking. Figure 8 displays the maximum temperature hold segments of each ground plane with different quantities of defects. Figure 8a is the same segment previously described in Figure 6b. Figure 8b displays the sensor response during the isothermal hold for the ground plane with minimal surface defects (defined as the surface with two initial saw cuts). The sensor response has the same downward frequency shift observed as the defect-free sensor but displays a decrease in magnitude as opposed to an increase. This does not agree with the simulation results which are shown in Figure 5b because this sensor experienced a spallation event (i.e., catastrophic fracturing of the oxide layer). Figure 8c displays the maximum temperature hold for the ground plane with maximum surface defects (defined as the surface with four initial saw cuts). The sensor response displays the same downward frequency shift as the other two ground planes but now very little S11 magnitude change. A distinctly different behavior was shown for each ground plane tested depending upon the relative population of crack formation and propagation.

**Figure 9:**
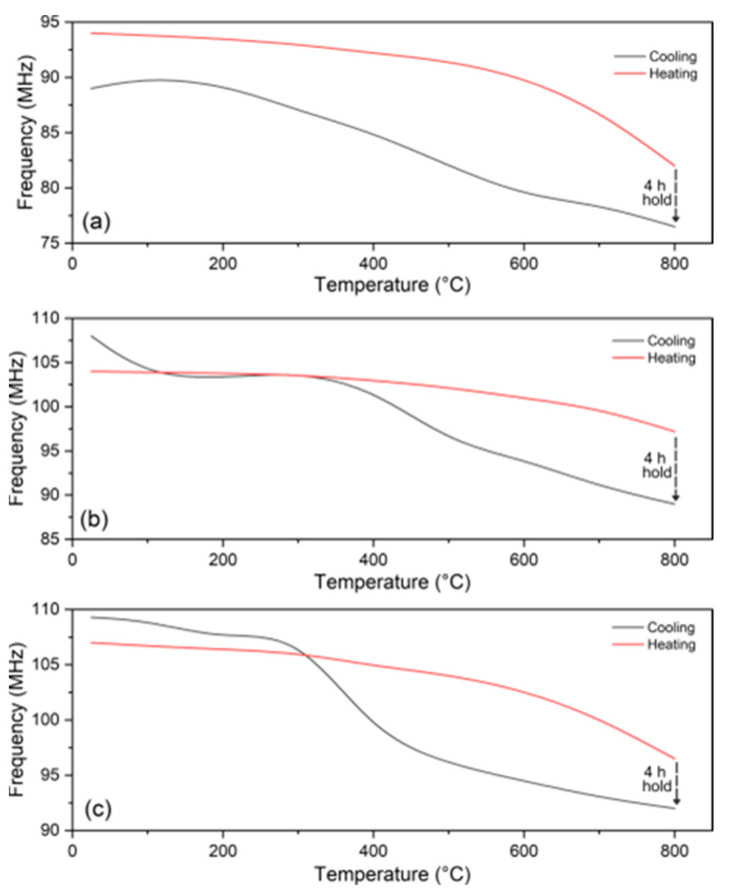
Passive wireless sensor frequency as a function of temperature during the heating and cooling stages of the experiment for the ground plane with different levels of pre-existing surface defects. (**a**) Defect-free ground plane. (**b**) Minimal defects in the ground plane. (**c**) Maximum defects in the ground plane.

Figure 9 displays the key sensor frequency versus temperature during the heating and cooling stages for the ground plane with different levels of pre-existing defects. Figure 9a displays the hysteresis of the defect-free ground plane, and it can be seen that the magnitude of the heating and cooling frequency shift is very consistent. A frequency shift occurs during the maximum temperature hold at 800 °C that causes a gap between the heating and cooling plots. This gap proves to be a mechanism for distinguishing spallation since both of the other ground planes observed in Figure 9b,c have a larger frequency change in their cooling rate. The cooling line crosses the heating line in both plots. This does not occur for the originally “defect-free” ground plane and is a result of spallation events for both defective ground plane levels.

These results relating to the irregular sensor response associated with surface defects assisted in better understanding other results discovered in the current work. Further sensor tests were completed on defect-free ground planes at temperatures <800 °C in this work. The sensors were tested also at 700 °C and 600 °C. The results of these experiments can be observed in Figures S5 and S6 (in the Supplementary Materials), respectively. In both of these temperature experiments, the sensors showed similar discontinuous responses during the thermal ramping stages (especially during the cooling stage); also, the same decrease in amplitude and frequency jumps were identified during the isothermal holds. From the previously discussed experiments, it may be concluded that the discontinuous jumps in the frequency and changes (decrease) in sensor amplitude should be associated with cracking and spallation events. This was found to be true, as when the samples were removed from the thermal chamber, the oxide layer was shown to be cracked or completely spalled from the Cu ground plane surface. Initially, the catastrophic failure of the oxide layer was found to be perplexing to the research team, since the Cu ground planes were all prepared with the same careful polishing techniques used for the initial 800 °C sample tests. Later, it was identified that below 800 °C a mixture of CuO and Cu_2_O phases formed during the thermal oxidation process, as shown by other researchers [45,46]. It was also shown by these researchers that the bonding strength of the oxide layer was lower than that at temperatures <800 °C and that the mixed-phase oxide would consistently crack and delaminate from the host Cu substrate [47]. Regardless, these results show the high potential of the given passive wireless sensor to monitor both the oxidation/corrosion rate of a metal at high temperatures as well as the formation and propagation of cracks within surface layers.

## 4. Conclusions

In this work, a planar passive LC resonator sensor was computationally designed using ANSYS HFSS modeling software. The software simulated the sensor performance trends accurately when a CuO layer (of varying thickness, representing a thermally growing oxide layer) was placed between the Cu ground plane and the wireless sensor. The simulation showed that a square five-turn planar Ag inductor supported on an Al_2_O_3_ substrate was the optimal design for corrosion characterization at 800 °C.

This sensor design was then fabricated using a screen-printing technique, where Ag particulate ink was printed and sintered on the Al_2_O_3_ plate. The sensor was placed on a polished Cu plate and operated at 800 °C for 4 h in air. The wireless signal (S11 parameter) was acquired by a Pt loop antenna in the near field using a VNA. Both the temperature and oxide growth were found to be precisely measured by the predictable shift in the target frequency. This work showed a similar correlated frequency shift and signal amplitude change with oxide growth as that previously demonstrated by a few other researchers for low-temperature experiments using commercial RFID tag sensors. In addition, for the first time, this work confirmed the oxide growth thickness with the frequency shift by directly characterizing the oxide thickness for the Cu 110 through SEM microstructural analysis and TGA kinetic studies. In addition, this work also proved the effect of oxide cracking and spallation events on the sensor response. This work purposely dictated these failure events by controlling the population of surface flaws on the metal surface (in this case, the ground plane for the sensor). These sensor experiments showed abrupt frequency jumps and a decrease in the signal amplitude, indicating catastrophic events. These results show that the simple sensor design will permit the wireless observation of corrosion thickness as well as an understanding of the crack population and spallation events occurring on the metal surface.

It must be noted though that the inconsistent nature of the cracking and spallation events adds complexity to the identification of oxide thickness (and even temperature) since the oxide thickness would need to be tracked and discontinuous movements (due to cracking) would need to be correlated to the predicted signal frequency trend (due to corrosion) to deconvolute each influence. Therefore, in the future, this sensor design will require extensive machine learning algorithms to deconvolute the temperature, corrosion (oxide growth), and cracking effects on the sensor response. In addition, future work may need to focus on alternative material sets for the inductor (outside the use of Ag) to further increase the applicability to higher temperatures and different environments. Also, work should center on testing the passive wireless corrosion sensors with more applicable high-temperature metals that are used in more aggressive environments. Overall, this technology could be invaluable in providing real-time corrosion data and assisting in predicting part/mechanism replacement schedules and/or critical failures, in a cost-effective and distributed manner.

## Figures and Tables

**Table 1:** Sensor simulation optimization parameters.

(a) Line Width (mm)	0.4	0.6	0.8	1.0	1.2	1.4	1.6
(b) Electrode height (µm)	20	30	40	50	60	70	80
(c) Line spacing (mm)	0.1	0.4	0.7	1.0	1.3	1.6	1.9
(d) Sensor scale (%)	55	70	85	100	115	130	145
(e) Inductor side number	3	4	5	6	7	8	circle
(f) Inductor turn number	1	2	3	4	5	6	7

## Data Availability

Data are contained within this article and the Supplementary Materials.

## Supplementary Materials

The following supporting information can be downloaded at: https://www.mdpi.com/article/10.3390/s24237806/s1, Table S1: Comparison of non-destructive corrosion measurement techniques [7,46,47]; Table S2: Guiding principles for ANSYS HFSS modeling [35]; Table S3: Properties of materials used in the ANSYS simulations; Table S4: General Workflow for Simulating the Sensor Design in ANSYS HFSS; Table S5: Calculated oxidation parabolic constants (k”) for Cu samples in g^2^ cm^−4^ s^−1^; Figure S1: Optical image of 5-turn sensor after bonding the Ag inductor pattern onto the Al_2_O_3_ substrate; Figure S2: Arrhenius plot for the oxidation of copper in different temperatures; Figure S3: Copper oxide thickness growth obtained in TGA curves during (a) the heating stage from room temperature to 800 °C, and (b) the temperature isothermal hold at 800 °C for 4 h; Figure S4: Graph of both the copper oxide thickness growth (extracted from the TGA data) and the measured sensor shift data, both graphed as a function of isothermal hold time at 800 °C; Figure S5: Passive wireless sensor heating and cooling results of the copper ground plane with no surface defects. (a) Sensor data for the heating stage from room temperature to 700 °C at a rate of 120 °C/h. (b) Sensor data at 700 °C isothermal hold for 4 h. (c) Sensor data for the cooling stage from 700 °C to room temperature at a rate of −120 °C/h; Figure S6: Passive wireless sensor heating and cooling results of the copper ground plane with no surface defects. (a) Sensor data for the heating stage from room temperature to 600 °C at a rate of 120 °C/h. (b) Sensor data at 600 °C isothermal hold for 4 h. (c) Sensor data for the cooling stage from 600 °C to room temperature at a rate of −120 °C/h.
