# Supplementary material for: Near-Field Passive Wireless Sensor for High-Temperature Metal Corrosion Monitoring
Source: Sensors (Basel). 2024 Dec 6;24(23):7806. doi: 10.3390/s24237806 (PMC11644951; doi:10.3390/s24237806)
Supplement: Supplementary file 1 [file sensors-24-07806-s001.zip › sensors-3284000-supplementary.pdf]

sensors

Supplementary

Table S1. Comparison of non-destructive corrosion measurement techniques [7][46][47].

Sensor Type	Performed In Situ	High-Temperature Monitoring	Accuracy	Operation Time	Advantages	Disadvantages
**Ultrasonic Sensor**	Yes	No	Medium	Short (minutes)	Non-invasive, depth measurement	Sensitive to surface roughness
**Pulsed Eddy Currents**	Yes	Yes	High	Medium (minutes)	Effective for subsurface defects	High cost, requires expertise
**Passive Wireless Sensors**	Yes	Yes	Medium	Long (continuous)	Low power consumption	Limited data collection speed
**Electrical Resistance Probe**	Yes	Yes	High	Medium (hours)	Immediate efficiency, easy installation	Valid only for specific areas
**Optical Fiber Sensors**	Yes	Yes	Very High	Short (minutes)	High precision, immune to electromagnetic interference	Fragility, requires special applications
**Corrosion Coupon**	No	Yes	Medium	Long (weeks/years)	Simple and low-cost	Slow response time, singular point measurement
**Electrochemical Sensor**	Yes	Yes	High	Short (minutes)	Quick response time, can measure various parameters	Requires precise calibration
**Magnetic Flux Leakage Sensor**	Yes	No	High	Medium (hours)	Detects subsurface defects	High cost, may require special training
**Electromagnetic Sensor**	Yes	No	Medium	Medium (minutes)	Fast response time, can detect various materials	Requires metallic field
**Pipeline Inspection Gauge**	No	No	Very High	Long (days)	High-precision, comprehensive data	Complex, high-cost

The software used in this work was ANSYS HFSS. As described on their website, ANSYS HFSS is a "3D electromagnetic (EM) simulation software for designing and simulating high-frequency electronic products such as antennas, antenna arrays, RF or microwave components, high-speed interconnects, filters, connectors, IC packages and printed circuit boards. Engineers worldwide use Ansys HFSS software to design high-frequency, high-speed electronics found in communications systems, advanced driver assistance systems (ADAS), satellites, and internet-of-things (IoT) products."

More information on the software, and its origin and scientific principles can be located on their website at:

https://www.ansys.com/products/electronics/ansys-hfss?utm_campaign=product&utm_medium=paid-search&utm_source=google&utm_content=digital_electronics_copr15el_contact_contact-us_hfss-electronics-brand-search_1a_en_global&campaignid=7013g000000cXBXAA2&utm_term=ansys%20hfss&gad_source=1&gclid=CjwKCAiA9IC6BhA3EiwAsbltOONHRB3qbGBYEAQLaCEXWkXKTBtCeZbmbwrLMhyE5qfsD2pwxNbdJRoCuAwQAvD_BwE#tab1-2

For those unfamiliar with this commercial software, the simulation software is based on a finite element method analysis (FEA) approach, similar to ANSYS's approach to mechanical property computational modeling packages. The element volume for the HFSS software is a tetrahedra, where these stacked tetrahedra will complete the mesh volume. The electric field solution for the designed mesh volume is completed by satisfying the inter-element boundary requirements defined by Maxwell's equations. A nice summary of the principles of the solutions is presented by ANSYS within Ref [35]. To save the reader some reference searching, the general description of the governing principles stated in this text is described as follows using the equations presented in Table S2.

As stated in Ref [35], the overarching goal of the calculation for the HFSS software is to solve for the electric field (E) using Eq. S1 and various excitation and boundary conditions defined by the user and the mesh developed. The software then calculates the corresponding magnetic field (H) using Eq. S2. As stated in this text, the main point is that the software calculates the E and M for each element volume. These volumes include conducting, dielectric, insulating, and magnetic materials, which include all the surrounding environment such as air or other conductors. To calculate for specific structures, such as the sensor in this work, the simulation software calculates a finite element matrix using the principal equations to find the fields and associated port S-matrix. The text (Ref. [35]) provides a nice procedure that the software generally follows, expressed as follows:

1) The structure is divided into a finite element mesh using tetrahedral elements.

2) The testing functions W_n, are defined into tetrahedrons, resulting in thousands of basis functions

3) The field is multiplied by the field Eq. S1 by a W_n and integrated over the solution volume which yields Eq. S3a

This procedure yields thousands of equations for $n=1,2,\ldots,N$

4) Manipulating the N equations, using Green's theorem and the divergence theorem yields Eq. S3b

for $n=1,2,\ldots,N$ writing, then this yields Eq. S4

5) Eq. S3b can then be rewritten as Eq. S5 for $n=1,2,\ldots,N$

6) Eq. S5 then has the form of Eq. S6 or as a matrix linear equation Eq. S7 as Ax=B

The N×N matrix is defined as A (which includes all the applied boundary conditions). B contains all the port excitations, voltage and current sources, and incident waves. The solution of B will result in the E field of all elements. It must be restated that this very concise description above comes from the review of the principles of this commercial software described by the manufacturer in Ref [35].

The workflow used by the researchers to complete the simulations of the sensor designs is presented in Table S3. This workflow can be followed by other researchers to replicate the presented results. If there are any questions or issues in repeating these results, then researchers should feel free to contact the authors of this work and they will be happy to assist.

Table S2: Guiding principles for ANSYS HFSS modeling [35].

Equations	Equation Number
$\nabla \times \left(\frac{1}{\mu_r} \nabla \times E\right) - k_0^2 \varepsilon_r E = 0$	S1
$H = \frac{j}{\omega\mu} \nabla \times E$	S2
$\int_V \left(W_n \cdot \nabla \times \left(\frac{1}{\mu_r} \nabla \times E\right) - k_0^2 \varepsilon_r W_n \cdot E\right) dV = 0$	S3a
$\int_V \left[(\nabla \times W_n) \cdot \frac{1}{\mu_r} \nabla \times E - k_0^2 \varepsilon_r W_n \cdot E\right] dV = \int_S (boundary\ terms)\, dS$	S3b
$E = \sum_m^N x_m W_n, n = 1,2 \ldots M$	S4
$\sum x_m \int_V \left[(\nabla \times W_n) \cdot \left(\frac{1}{\mu_r} \nabla \times W_m\right) - k_0^2 W_n \cdot W_m\right] dV = \int_S (boundary\ terms)\, dS$	S5
$\sum x_m A_{n,m} = b_n, n = 1,2 \ldots, N$	S6
$Ax = b$	S7

Table S3. Properties of materials used in the ANSYS simulations.

	Relative Permittivity	Relative Permeability	Conductivity (S/m)
Silver	1.000	0.999	6.1×10^{7}
Platinum	1.000	1.000	9.3×10^{6}
Copper	1.000	0.999	5.8×10^{6}
Air	1.001	1.000	0
Copper Oxide	12.00	1.0	1
Alumina (25 °C)	9.800	1.000	1×10^{-4}
Alumina (800 °C)	11.00	1.000	1×10^{-4}

Table S4. General Workflow for Simulating the Sensor Design in ANSYS HFSS.

Step	Description
1	Open a new project file in ANSYS.
2	Create a 3D box with dimensions matching the steel plate. Assign steel as the material for the box.
3	Add a second box on top of the steel plate to represent the dielectric material (alumina). Assign alumina as the material.
4	On the top surface of the dielectric material, create the sensor geometry based on the dimensions listed in Table S2.
5	Draw a line to form a loop antenna. Assign platinum as the material for the antenna.
6	At the two ends of the antenna, create a sheet to connect them, forming a "port" required for the lumped port setup.
7	Select the ends of the antenna, assign excitation, and define the setup as a Lumped Port.
8	Create a radiation box in the viewing window to encapsulate all components. Assign air as the material for this box.
9	Set up the simulation by choosing the Autotype and defining the frequency sweep range and number of data points.
10	Validate the model to ensure correctness, then simulate the setup to perform frequency analysis on the sensor.
11	Analyze the results, specifically focusing on the S11 parameter.

Table S5. Calculated oxidation parabolic constants (k'') for Cu samples in $g^2\ cm^{-4}\ s^{-1}$.

Temperature	k'' ($g^2\ cm^{-4}\ s^{-1}$)
600 °C	2×10^{-8}
700 °C	5×10^{-8}
800 °C	1×10^{-7}

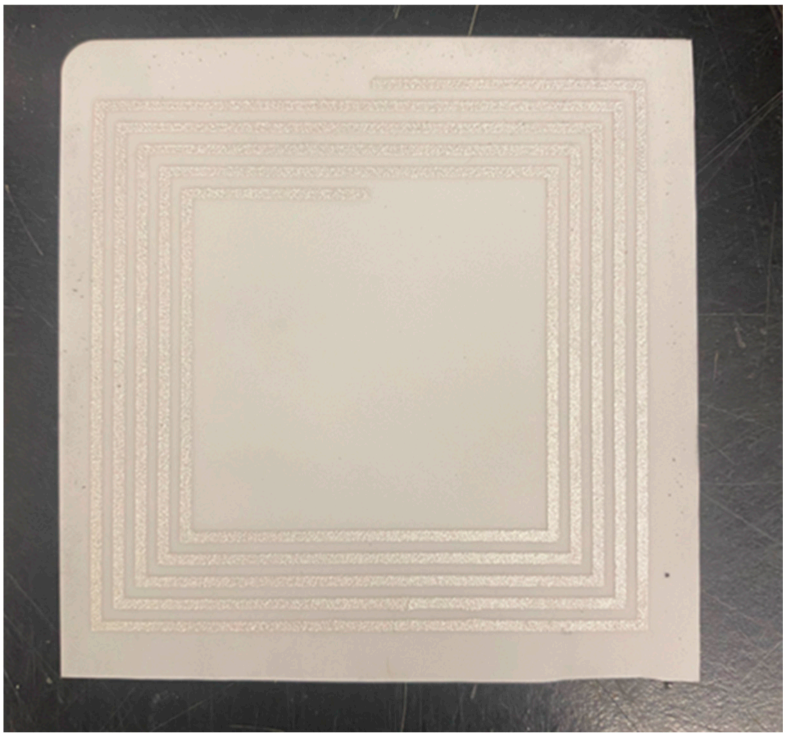

Figure S1. Optical image of 5-turn sensor after bonding the Ag inductor pattern onto the Al_2O_3 substrate.

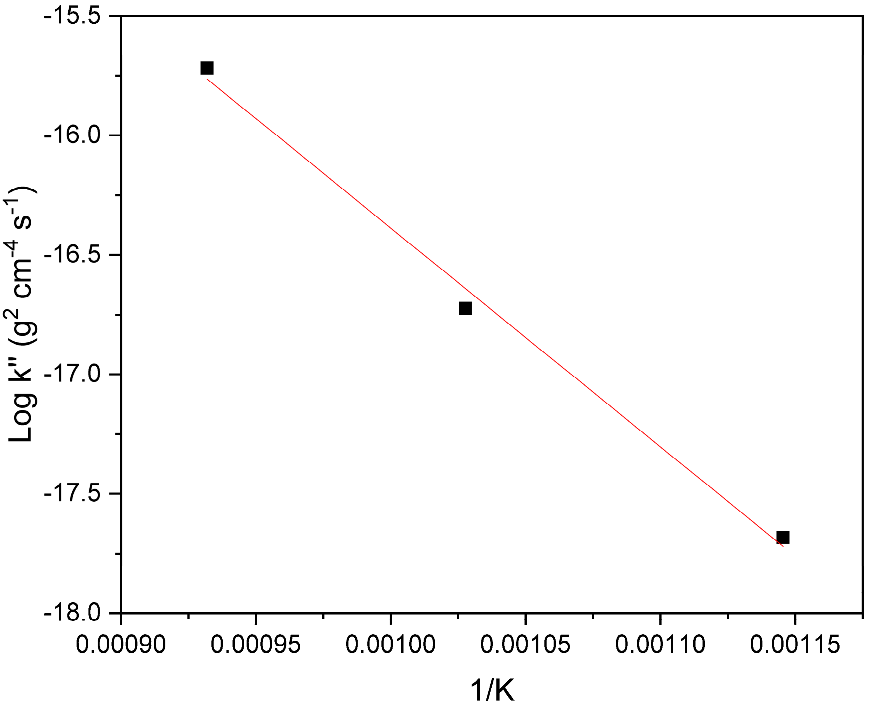

Figure S2. Arrhenius plot for the oxidation of copper in different temperatures.

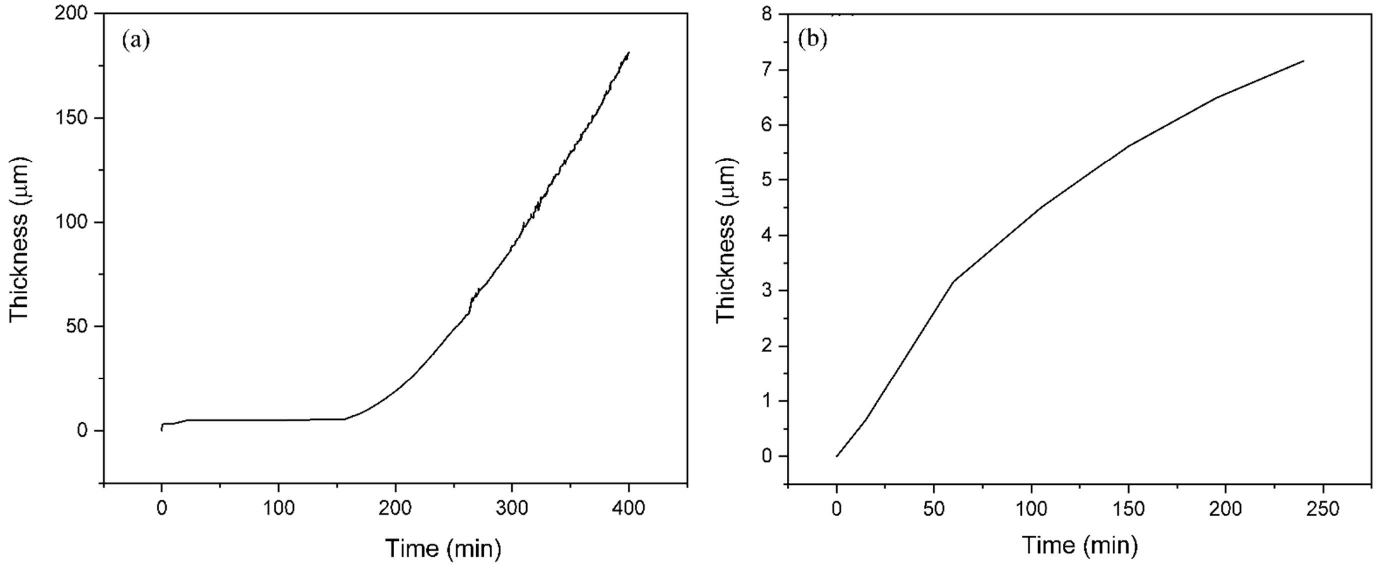

Figure S3. Copper oxide thickness growth obtained in TGA curves during (a) the heating stage from room temperature to 800 °C, and (b) the temperature isothermal hold at 800 °C for 4 h.

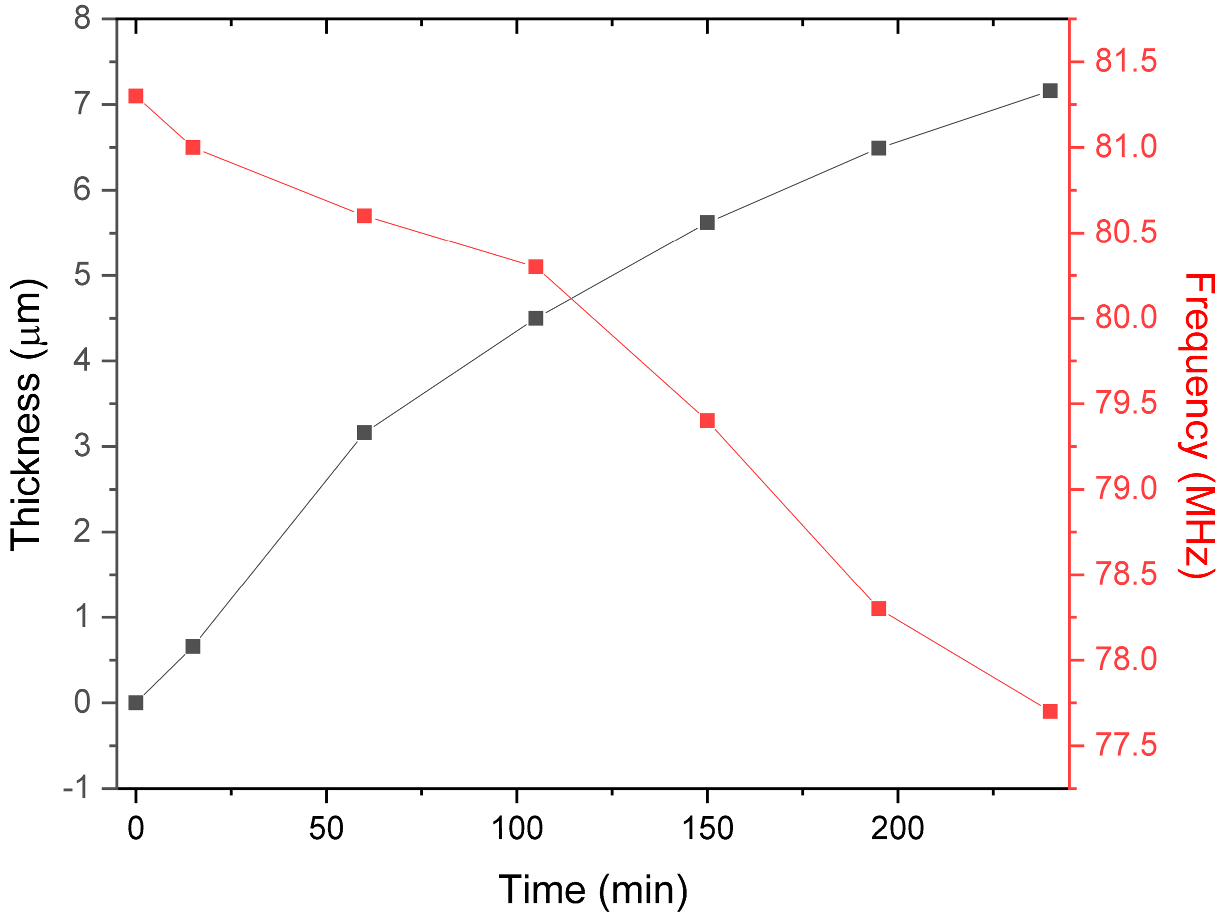

Figure S4. Graph of both the copper oxide thickness growth (extracted from the TGA data) and the measured sensor shift data, both graphed as a function of isothermal hold time at 800 °C.

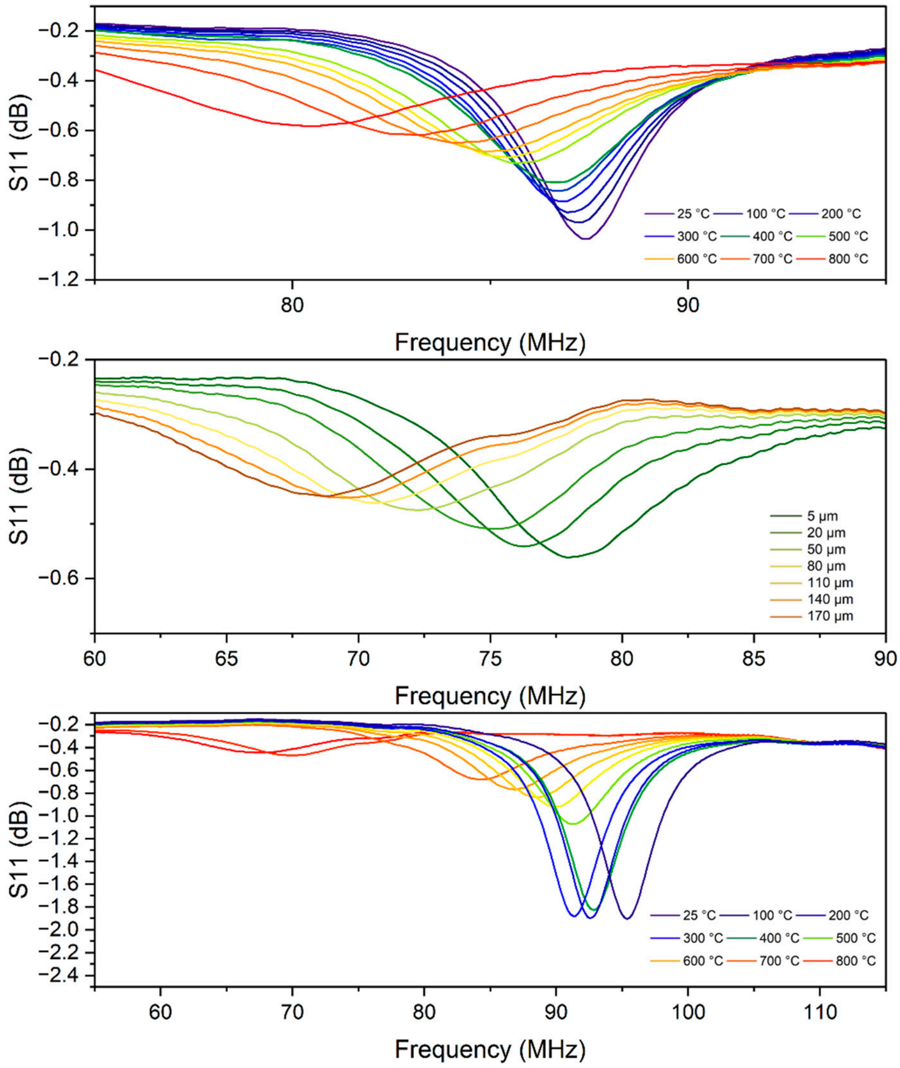

Figure S5. Passive wireless sensor heating and cooling results of the copper ground plane with no surface defects. **(a)** Sensor data for the heating stage from room temperature to 700 °C at a rate of 120 °C/h. **(b)** Sensor data at 700 °C isothermal hold for 4 h. **(c)** Sensor data for the cooling stage from 700 °C to room temperature at a rate of −120 °C/h.

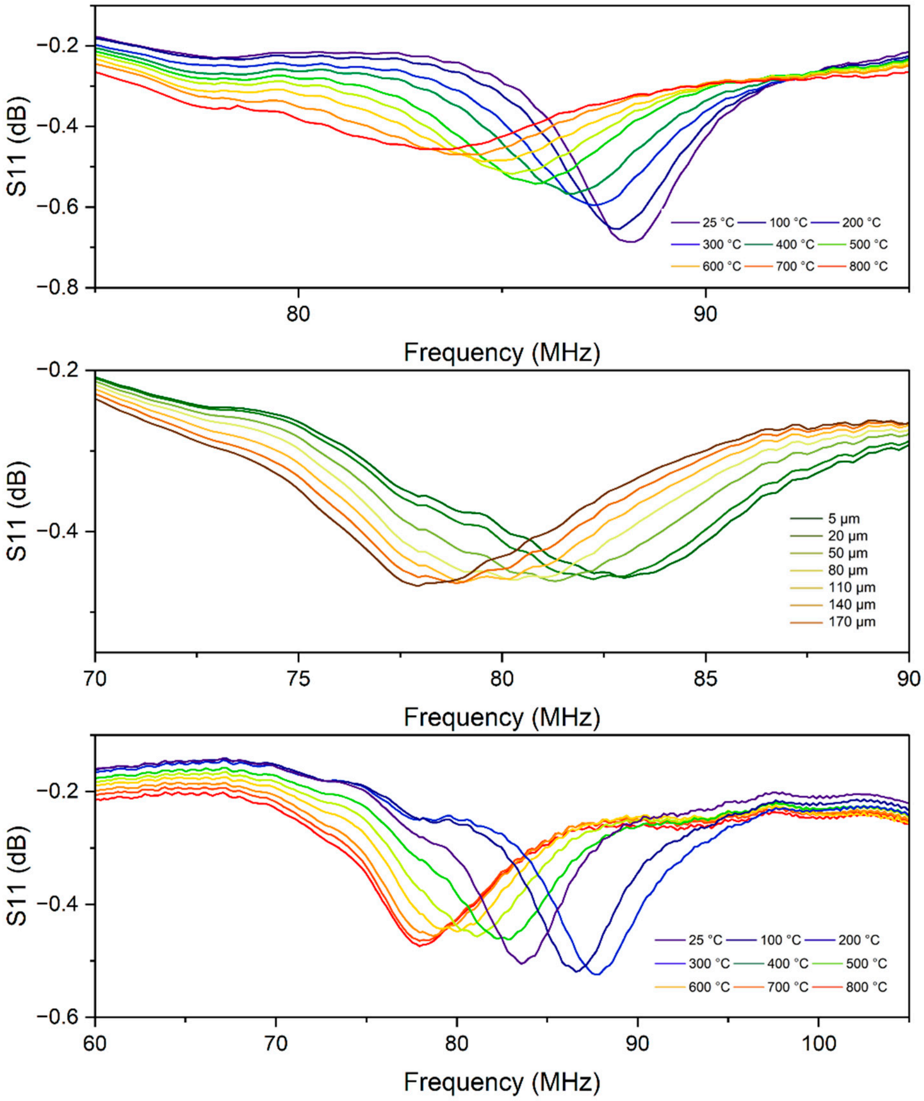

Figure S6. Passive wireless sensor heating and cooling results of the copper ground plane with no surface defects. **(a)** Sensor data for the heating stage from room temperature to 600 °C at a rate of 120 °C/h. **(b)** Sensor data at 600 °C isothermal hold for 4 h. **(c)** Sensor data for the cooling stage from 600 °C to room temperature at a rate of −120 °C/h.
